# Auditory, Visual, and Cross-Modal Mismatch Negativities in the Rat Auditory and Visual Cortices

**DOI:** 10.3389/fnhum.2021.721476

**Published:** 2021-09-17

**Authors:** Tomoyo Isoguchi Shiramatsu, Kanato Mori, Kotaro Ishizu, Hirokazu Takahashi

**Affiliations:** ^1^Graduate School of Information Science and Technology, The University of Tokyo, Tokyo, Japan; ^2^Institute for Quantitative Biosciences, The University of Tokyo, Tokyo, Japan

**Keywords:** cross-modal information processing, deviance detection, mismatch negativity, microelectrode array, sensory cortex

## Abstract

When the brain tries to acquire an elaborate model of the world, multisensory integration should contribute to building predictions based on the various pieces of information, and deviance detection should repeatedly update these predictions by detecting “errors” from the actual sensory inputs. Accumulating evidence such as a hierarchical organization of the deviance-detection system indicates that the deviance-detection system can be interpreted in the predictive coding framework. Herein, we targeted mismatch negativity (MMN) as a type of prediction-error signal and investigated the relationship between multisensory integration and MMN. In particular, we studied whether and how cross-modal information processing affected MMN in rodents. We designed a new surface microelectrode array and simultaneously recorded visual and auditory evoked potentials from the visual and auditory cortices of rats under anesthesia. Then, we mapped MMNs for five types of deviant stimuli: single-modal deviants in (i) the visual oddball and (ii) auditory oddball paradigms, eliciting single-modal MMN; (iii) congruent audio-visual deviants, (iv) incongruent visual deviants, and (v) incongruent auditory deviants in the audio-visual oddball paradigm, eliciting cross-modal MMN. First, we demonstrated that visual MMN exhibited deviance detection properties and that the first-generation focus of visual MMN was localized in the visual cortex, as previously reported in human studies. Second, a comparison of MMN amplitudes revealed a non-linear relationship between single-modal and cross-modal MMNs. Moreover, congruent audio-visual MMN exhibited characteristics of both visual and auditory MMNs—its latency was similar to that of auditory MMN, whereas local blockage of *N*-methyl-D-aspartic acid receptors in the visual cortex diminished it as well as visual MMN. These results indicate that cross-modal information processing affects MMN without involving strong top-down effects, such as those of prior knowledge and attention. The present study is the first electrophysiological evidence of cross-modal MMN in animal models, and future studies on the neural mechanisms combining multisensory integration and deviance detection are expected to provide electrophysiological evidence to confirm the links between MMN and predictive coding theory.

## Introduction

Prediction is an essential brain function required to understand the surrounding environment correctly. According to many theories, including Bayesian and Kahneman’s frameworks, the brain is thought to build predictions from various types of information and update these repeatedly by observing “errors” to acquire an elaborate model of the external world (Kahneman, 2011; Clark, 2013). In this prediction-building process, multisensory integration is thought to play an important role to obtain meaningful perceptual experiences by integrating information from different sensory modalities (Tononi et al., 1998; Alais et al., 2010). The latter “updating” process is thought to be triggered by a “prediction error,” recognized by using the deviance-detection system of the brain. A recent report that prediction error is hierarchically represented as deviance-detecting neural activities along the sensory pathway is in accordance with the hierarchical predictive coding framework (Friston, 2005; Stefanics et al., 2014). Additionally, previous studies demonstrating that deviance-detecting activities reflect experience and learning suggest that the deviance-detection system is deeply involved in predictions mediated by internal models of the brain, as the predictive coding framework suggests (Menning et al., 2002; Shiramatsu and Takahashi, 2018). Therefore, combination of the multisensory integration and deviance-detecting system contribute to the brain building, maintaining, and renewing a model of the external environment.

Many studies have focused on the deviance detection system of the brain, primarily because the leading candidate for its neural correlates, that is, mismatch negativity (MMN), was discovered relatively early. The first paradigm designed to observe MMN was developed for the auditory domain—an infrequent or deviant sound following a frequent or standard sound elicits auditory MMN (aMMN) (Näätänen et al., 1978). Later, MMN was also confirmed in the context of other sensory modalities (Kekoni et al., 1997; Musall et al., 2017). Currently, visual MMN (vMMN) is the second most prominent focus among MMN studies, particularly in humans (Pazo-Alvarez et al., 2003). Many previous studies have demonstrated that both aMMN and vMMN cannot be fully explained by adaptation, and unpredictable deviations from abstract rules can also elicit MMN (Czigler et al., 2002, 2006; Pazo-Alvarez et al., 2004; Astikainen and Hietanen, 2009; Kimura et al., 2009; Chang et al., 2010; Clifford et al., 2010; Stefanics and Czigler, 2012; Czigler, 2014). This deviance-detection property of MMN has stimulated a predictive coding framework that considers MMN as a type of prediction-error signal (Friston, 2005; Garrido et al., 2008, 2009; Den Ouden et al., 2012). Together with the fact that integration of visual and auditory information is essential for object recognition, the elucidation of the relationship between multisensory integration and MMN should enhance the theoretical understanding of predictive coding.

Despite its importance, very few studies have investigated how cross-modal information processing affects MMN. One reason for this is that the primary brain areas focused on in studies of MMN and multimodal integration are different. The sensory cortex is the earliest source of MMN (Scherg et al., 1989; Csépe et al., 1992; Tiitinen et al., 1993; Alho et al., 1996; Berti and Schröger, 2001; Pincze et al., 2001; Czigler et al., 2002; Shiramatsu et al., 2013), whereas the parietal and frontal cortices are assumed to be essential for multisensory integration (Calvert, 2001; Sereno and Huang, 2014). Another reason for the paucity of these studies is the difficulty in the experimental control of top-down effects, such as prior knowledge and attention. Most human studies investigating the cross-modal effect on MMN have utilized audio-visual illusions, such as the McGurk–MacDonald illusion and the ventriloquist illusion, which often depend on linguistic knowledge (Colin et al., 2002a, b; Stekelenburg et al., 2004; Saint-Amour et al., 2007; Froyen et al., 2008; Andres et al., 2011; Stekelenburg and Vroomen, 2012). Additionally, it is difficult to exclude the influence of attention on the cross-modal information processing when evaluating these illusions using linguistic stimuli. However, notwithstanding the difficulties involved, controlling these top-down effects is important when attempting to clarify the “pre-attentive” cross-modal effects on MMN.

To address this challenge, the present study used anesthetized rats as the first animal model to be used in studying cross-modal MMN. Accumulating evidence has indicated that both aMMN and vMMN in rodents exhibit characteristics similar to those in humans (Shiramatsu et al., 2013; Hamm and Yuste, 2016). Moreover, the top-down effects of prior knowledge and attention can be minimized by using simple non-linguistic stimuli and anesthesia, respectively. Thus, these controls would help reveal the most primitive cross-modal effect on MMN. We also developed a new microelectrode array to cover both the visual and auditory cortices of rats for mapping vMMN, aMMN, and audio-visual MMN. We first tested the deviance-detection property of vMMN because it has not been demonstrated in rats. We then investigated cross-modal effects on MMN by comparing the amplitudes and latencies of the single-modal and audio-visual MMNs. Lastly, we locally blocked *N*-methyl-D-aspartic acid (NMDA) receptors in the visual cortex to investigate the neural mechanisms of cross-modal MMN.

## Materials and Methods

This study was conducted in strict accordance with the “Guiding Principles for the Care and Use of Animals in the Field of Physiological Science” published by the Japanese Physiological Society. The experimental protocol was approved by the Committee on the Ethics of Animal Experiments at the Graduate School of Information Science and Technology, the University of Tokyo (Permit Number: JA20-2). All surgeries were performed under isoflurane anesthesia, and all efforts were made to minimize the suffering of animals. After the experiments, the animals were euthanized with an overdose of pentobarbital sodium (160 mg/kg, i.p.). The raw data supporting the conclusions of this manuscript will be made available by the authors, without undue reservation, to any qualified researcher.

### Implantation of the Head-Fix Attachment

**FIGURE 1:**
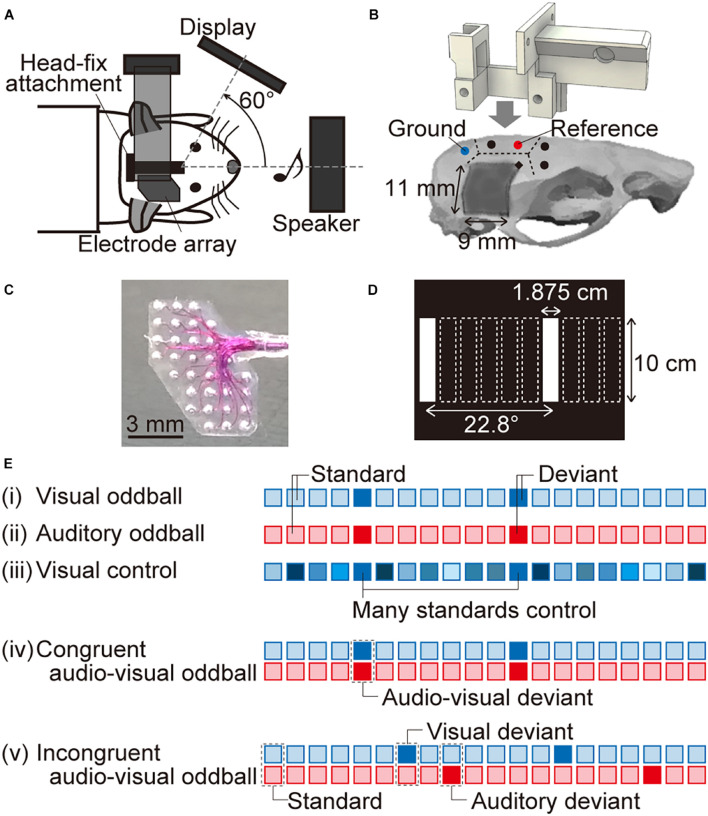
Experimental setup. **(A)** Schema of the experimental system. A custom-designed head-fix attachment implanted on the skull of each tested animal was used to fix it to the experimental neural recording system. The animals were anesthetized using isoflurane, the right visual and auditory cortices were exposed, and an electrode array was positioned onto the surface of the brain. Visual and auditory stimuli were presented from a display monitor facing an axis 60° left from the sagittal axis and from a speaker in front of the rat, respectively. **(B)** The dotted lines indicate the boundaries of the skull. The five circles indicate where the screws (diameter, 1 mm; length, 3 mm) were anchored. Two of these screws made electrical contact with the dura mater for use as the ground and reference electrodes (indicated by the blue and red circles, respectively). The head-fix attachment was fixed on the skull using dental resin. For neural recording, we started drilling into the skull from the point indicated by a black diamond (2-mm posterior and 1.5-mm lateral to the bregma) and removed a part of the right temporal skull (approximately 11 mm × 9 mm, dark gray color). **(C)** Magnified image of the surface microelectrode array with 32 recording sites. The recording sites in the upper left and bottom right cover the visual and auditory cortices, respectively. **(D)** In the visual “many standards control” paradigm, white vertical bars (1.875-cm wide and 10-cm high) at 10 different horizontal positions were displayed on the monitor. Two of these positions (i.e., the first and seventh from the left) were also presented in the oddball paradigm. **(E)** We tested five paradigms to record visual and auditory MMNs. The blue and red squares indicate visual and auditory stimuli, respectively. In (i) the visual oddball and (ii) the auditory oddball paradigm, standard and deviant stimuli were randomly delivered at a 90 and 10% frequency, respectively. (iii) In the visual control paradigm, 10 visual stimuli at different horizontal positions were presented randomly with the same probability as the deviant in the oddball paradigm, i.e., 10%. We also tested two audio-visual oddball paradigms: (iv) in the congruent audio-visual oddball paradigm, visual and auditory deviant stimuli were always presented together, while (v) in the incongruent audio-visual oddball paradigm, they were delivered independently. When the visual and auditory deviant stimuli were presented together in the incongruent audio-visual oddball paradigm, we eliminated the corresponding responses from the analysis. We also performed a second recording for the four oddball paradigms, in which we converted the standard and deviant stimuli to quantify MMN by comparing standard and deviant responses for the same stimuli.

Eleven male Wistar rats (postnatal weeks 9–17; body weight, 260–360 g) were used for the experiments. The rats were first implanted with a custom-made head-fix attachment (Figures 1A,B), which was used to fix them to the experimental neural recording setup. Briefly, the animals were anesthetized using isoflurane (Mylan Inc., PA, United States; 5% v/v in air for induction and 2–3.5% for maintenance) and were held in a stereotaxic apparatus (SR-50; Narishige Group, Tokyo, Japan). Thereafter, a skin incision was made under local anesthesia using xylocaine (1%, 0.2 ml; Aspen Japan, Tokyo, Japan) to expose part of the skull. Five screws (diameter, 1 mm; length, 3 mm) were anchored to the skull—two in the left parietal bone, two in the frontal bone, and one in the interparietal bone (Figure 1B). Wires were connected to two of the screws for use as the reference and ground electrodes. Specifically, the screw in the interparietal bone, in contact with the dura over the cerebellum, was used as the ground electrode, and the frontal screw in the left parietal bone, in contact with the dura over the left somatosensory cortex, was used as the reference electrode (the blue and red circles, respectively, in Figure 1B). Several previous studies have reported auditory evoked potentials (AEPs) and aMMN using a reference electrode on the somatosensory cortex (Shiramatsu et al., 2013; Shiramatsu and Takahashi, 2018). The screws were fixed to the skull using a dental adhesive (Super-Bond C&B; Sun Medical Co., Ltd., Shiga, Japan), after which the head-fix attachment was mounted and fixed on the skull using dental resin (Unifast TRAD; GC Corporation, Tokyo, Japan). The attachment was custom designed and 3D printed using acrylonitrile butadiene styrene plastic. Part of the right parietal and right temporal bones were covered using dental silicone (DentSilicone-V; Shofu Inc., Kyoto, Japan) rather than dental resin and sealed until we removed these bones at the time of neural recording. After the implantation procedure, an anti-inflammatory agent (Capisten; 5 mg/mL, 0.2 mL; Kissei Pharmaceutical Co., Ltd., Nagano, Japan) and an antibiotic (Bixillin; 25 mg/mL, 0.2 mL; Meiji Seika Pharma Co., Ltd., Tokyo, Japan) were injected intramuscularly to avoid infection.

### Neural Recording

More than 3 days after the implantation of the head-fix attachment, the rats were anesthetized again using isoflurane (5% v/v in air for induction and 1–3.5% for maintenance) and held in place in the experimental setup for neural recording (Figure 1A). The dental silicone was removed, and the right temporal muscle, cranium, and dura overlying the visual and auditory cortices were locally anesthetized using xylocaine (1%, 0.1–0.3 mL). The exposed cortical surface was perfused with saline to prevent desiccation, and the cisternal cerebrospinal fluid was drained to minimize cerebral edema. A heating blanket was used to maintain body temperature at approximately 37°C. The respiration rate, heart rate, and hind-paw withdrawal reflexes were monitored throughout the experiment to ensure that an adequate and stable level of anesthesia was maintained.

A surface microelectrode array (Figure 1C; TU218-008; Unique Medical Co., Ltd., Tokyo, Japan) with 32 recording sites simultaneously recorded visual evoked potentials (VEPs) and AEPs from the visual and auditory cortices, respectively. This microelectrode array covered an area of 5 mm × 7 mm, with the recording sites in the upper left and bottom right quadrants, covering the visual and auditory cortices, respectively. The recording sites were made of platinum and placed between two silicon rubber sheets at a center-to-center distance of 1 mm. The diameter of the exposed surface of each recording site was 250 μm. Neural signals were obtained with an amplification gain of 1,000, a digital filter bandpass of 0.3–500 Hz, and a sampling frequency of 1 kHz (Cerebus Data Acquisition System; Blackrock Microsystems LLC, Salt Lake City, UT, United States).

### Visual and Acoustic Stimulation

Visual and acoustic stimuli were provided using MATLAB (MathWorks, Natick, MA, United States) and Psychtoolbox.^1^ A display monitor (LCM-T102AS; Logitec Corp., Tokyo, Japan) was positioned 20 cm from the left eye of the animal, at an axis of 60° left from the sagittal axis. A speaker (ST400 BLK; JBL Professional, Northridge, CA, United States or DLS-108X; Alpine Electronics Inc., Tokyo, Japan) was positioned 15 cm in front of the animal. Acoustic stimuli were calibrated at the pinna using a 1/4-inch microphone (4939; Brüel & Kjær, Nærum Denmark) and a spectrum analyzer (CF-5210; Ono Sokki Co., Ltd., Yokohama, Japan). The stimulus level was presented in terms of the sound pressure level in decibels with respect to 20 μPa [dB sound pressure level (SPL)]. The order of data acquisition was randomized, although not completely.

First, we recorded flash-elicited VEPs and click-elicited AEPs to demonstrate that the microelectrode array could separately map the neural activities in the visual and auditory cortices. A flash was a white circle with a 7.5-cm radius on a black background for a duration of 400 ms, and a click was a monophasic positive wave with a duration of 10 ms or 50 μs. The inter-onset interval was 900 ms, and the stimuli were separately presented 60 or 100 times. The amplitude of the middle-latency response, i.e., visual P1 (vP1) or auditory P1 (aP1), was quantified as the maximum potential within 200 ms from the onset of the stimulus.

Single-modal MMN (vMMN and aMMN) and cross-modal MMN were then obtained using several oddball paradigms. The visual test stimuli were white vertical bars (1.875-cm wide and 10-cm high) against a black background, presented for a duration of 400 ms. The bars appeared in two different horizontal positions, 22.8° apart on the viewing angle (first and seventh from the left in Figure 1D). The auditory test stimuli were tone bursts (8 or 16 kHz, 60 dB SPL) for a duration of 400 ms, including 5-ms rise/fall times. In the visual or auditory oddball paradigm (Figures 1Ei,ii), the two white bars or two pure tones served as either a frequent standard (*p* = 0.9) or a rare deviant (*p* = 0.1). The inter-onset interval between stimuli was 900 ms. After we obtained 60 or 100 deviant responses, we swapped the test position or test frequency of standard and deviant stimuli and then delivered the second oddball session. The grand-averaged deviant response was subtracted from the standard response to the same stimuli, i.e., the position of the bar and tone frequency and the MMN amplitude was quantified as the maximum potential of this difference wave between 50 and 450 ms from the onset of the stimulus. The latency of MMN was also obtained as the post-stimulus time when the amplitude of the MMN was quantified as the maximum potential difference.

To test whether vMMN in rats exhibited deviance detection properties, VEPs were also investigated in the “many standards control” paradigm (Figure 1Eiii). In this control paradigm, white bars in 10 different horizontal positions, including two stimuli used in the oddball paradigm, were presented randomly (Figure 1D). The inter-onset interval was 900 ms. The probability of appearance of each test stimulus was identical to that of the deviant stimuli, i.e., 10%, and 60 or 100 control responses were obtained.

To test cross-modal MMN, we delivered two types of cross-modal oddball paradigms, i.e., the congruent and incongruent audio-visual oddball paradigms (Figures 1Eiv,v). In these paradigms, the inter-onset interval was same as that in the single-modal oddball paradigms, i.e., 900 ms, and standard stimulus was a combination of the bar at the first position from the left (“left bar”) and the 8-kHz tone burst (“low tone”) in the first oddball session, or the bar at the seventh position from the left (“right bar”) and the 16-kHz tone burst (“high tone”) in the second oddball session. In the congruent paradigm, visual and auditory deviants were always presented together; therefore, the deviant stimulus was a combination of “right bar” and “high tone” in the first congruent oddball session. From this paradigm, we obtained the amplitude and the latency of audio-visual MMN (avMMN) in the same way as the single-modal MMN. In the incongruent paradigm, visual and auditory deviants were independently presented; therefore, the stimulus with visual deviant was a combination of “right bar” and “low tone,” and the stimulus with auditory deviant was a combination of “left bar” and “high tone” in the first incongruent oddball session with the standard stimuli of “left bar” and “low tone.” When the visual and auditory deviants were unexpectedly delivered together in the incongruent audio-visual oddball paradigm, we eliminated the corresponding responses from the analysis. To quantify the amplitude and latency of MMN in the incongruent oddball paradigm, the grand-averaged deviant response was subtracted from the standard response in the other session; specifically, the visual-deviant response for “right bar” and “low tone” was subtracted from the standard response for “right bar” and “high tone.” Moreover, the auditory-deviant response for “left bar” and “high tone” was subtracted from the same standard response. The amplitude and latency of MMN were then obtained from the same post-stimulus time as the single-modal MMN.

### Administration of an NMDA Receptor Antagonist

To investigate whether NMDA receptors in the visual cortex mediate vMMN and cross-modal MMN, MMNs were also measured following the direct administration of the NMDA receptor agonist D-(-)-2-amino-5-phosphonopentanoic acid (AP5) onto the surface of the visual cortex. Briefly, after the first recording under the oddball paradigms and control paradigm, we removed the microelectrode array and placed a 2% (20 g/L) agarose gel sheet containing 100 μM AP5 onto the surface of the visual cortex. The auditory cortex was covered with a piece of cotton soaked in saline solution to prevent AP5 infiltration. After 15 min, we removed the gel sheet and cotton, mounted the surface array, and recorded MMNs under the auditory oddball, visual oddball, and congruent audio-visual oddball paradigms.

### Statistical Analysis

To confirm separate mapping from the visual and auditory cortices, multiple comparisons between the putative regions were conducted separately for vP1 and aP1 using the Kruskal-Wallis test. For *post hoc* comparison, the Wilcoxon one-sided signed-rank test with Bonferroni correction for three comparisons was used.

To demonstrate adaptation for the repetitive standard stimuli, we compared vP1 for the standard, deviant, and “many standards control” VEPs using the Kruskal-Wallis test for multiple comparisons and the Wilcoxon one-sided signed-rank test with Bonferroni correction for three comparisons as a *post hoc* test. Additionally, the Wilcoxon one-sided signed-rank test was used to investigate the deviance-detection property of vMMN by comparing the amplitude of negative deflections between the subtraction of deviant responses from the standard or control response.

To test the cross-modal effect on MMN, comparisons of MMN amplitude were assessed. The Wilcoxon one-sided signed-rank test was applied to compare (1) amplitude of vMMN in the single-modal visual oddball and amplitude of MMN for the visual deviance in the incongruent oddball, (2) amplitude of aMMN in the single-modal auditory oddball and amplitude of MMN for the auditory deviance in the incongruent oddball, and (3) amplitude of avMMN in the congruent oddball and the summation of the amplitude of vMMN and aMMN. Additionally, to reveal the propagation of MMN, we compared the latency of each MMN between the visual and auditory cortices.

Finally, to assess effect of the blockade of NMDA receptors in the visual cortex, the Wilcoxon one-sided signed-rank test was applied to compare the amplitude of each MMN before the blockade with that after the blockade.

All statistical analyses were performed using MATLAB (MathWorks).

## Results

### Mapping of the Evoked Potentials in the Visual and Auditory Cortices

**FIGURE 2:**
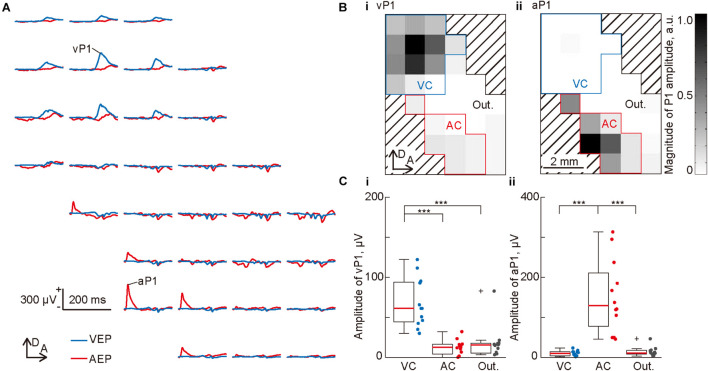
Mapping of the evoked potentials in the visual and auditory cortices. **(A)** Representative mapping of the waveform of a flash-elicited visual evoked potential (VEP; blue lines) and click-elicited auditory evoked potential (AEP; red lines) recorded simultaneously from 32 recording sites. Each waveform is approximately aligned in the spatial coordinates of the recording sites of the surface microelectrode array. Amplitudes of the visual P1 (vP1) and auditory P1 (aP1) was quantified as the maximum amplitude within 200 ms from the onset of the stimulus. **(B)** Spatial distributions of (i) vP1 and (ii) aP1. The gray level at each grid corresponds to the P1 amplitude at each recording site. The recording sites surrounded by blue or red lines were categorized as the putative visual cortex (VC) or auditory cortex (AC), which showed a vP1 or aP1 amplitude larger than 10% of the maximum amplitude. The other recording sites were categorized as the outer region. **(C)** Regional differences in (i) vP1 and (ii) aP1. Dots indicate the mean amplitudes of vP1 and aP1 among each putative region in individual animals (*n* = 12 animals). Asterisks indicate statistical significance in the *post hoc* analysis: ***p < 0.001 (Wilcoxon one-sided signed-rank test with Bonferroni correction for three comparisons, following the Kruskal-Wallis test).

Figure 2A shows the representative cortical mapping of flash-elicited VEPs and click-elicited AEPs. Both VEPs and AEPs exhibited clear positive potentials, i.e., vP1 and aP1, and aP1 exhibited shorter latencies than vP1. We quantified the amplitude of these P1s as the maximum amplitude within 200 ms from the onset of the stimulus, then mapped them. As shown in these maps (Figure 2B), vP1 and aP1 had separate activation foci, which seemed to be localized in the visual and auditory cortices, that is, the upper and lower parts of the recording area, respectively. Based on this observation, we putatively divided the recording area into three regions: the visual cortex, including 15 or fewer recording sites showing a vP1 amplitude larger than 10% of the maximum amplitude among all the recording sites; the auditory cortex, including 10 or fewer recording sites showing an aP1 amplitude larger than 10% of the maximum amplitude among all recording sites; and the outer region, which encompassed the remaining recording sites (Figure 2B). Consequently, the mean amplitude of P1s in these areas was significantly different. The multiple comparison and *post hoc* analyses showed that vP1 was larger in the putative visual cortex (Figure 2Ci; Kruskal-Wallis test, *p* = 5.6 × 10^–6^; *post hoc* Wilcoxon one-sided signed-rank test with Bonferroni correction for three comparisons, *p* = 0.00024 for visual cortex vs. auditory cortex, and visual cortex vs. outer region, respectively), and that aP1 was larger in the putative auditory cortex (Figure 2Cii; Kruskal-Wallis test, *p* = 5.6 × 10^–6^; *post hoc* Wilcoxon one-sided signed-rank test with Bonferroni correction for three comparisons, *p* = 0.00024 for auditory cortex vs. visual cortex, and auditory cortex vs. outer region, respectively). These results suggest that the surface microelectrode array could separately map the evoked responses in these cortical regions. Thus, we continued to adopt these putative visual and auditory regions in the subsequent analyses.

### Deviance-Detecting Property of the vMMN

**FIGURE 3:**
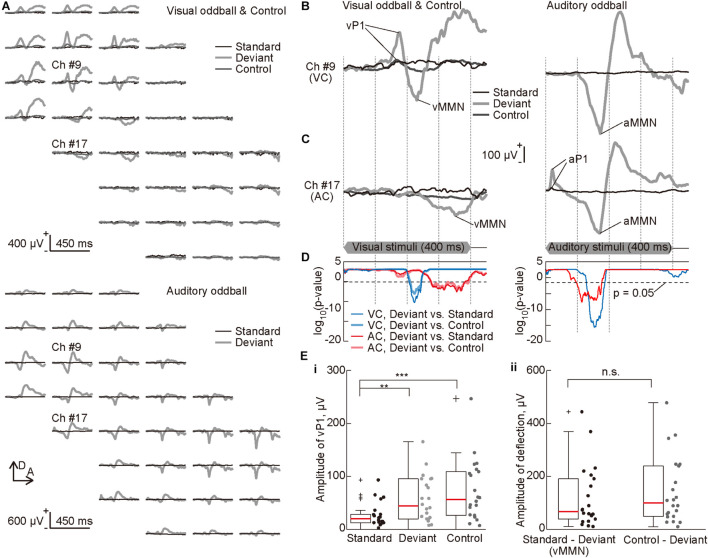
Deviance detection properties of visual mismatch negativity (vMMN). **(A)** Representative mapping of visual evoked potential (VEP) recorded in the visual oddball and control paradigms (top) and auditory evoked potential (AEP) recorded in the auditory oddball paradigm (bottom). The standard (black lines), deviant (bold light gray lines), and the control (dark gray lines) responses were approximately aligned in the spatial coordinates of the recording sites of the surface microelectrode array. **(B,C)** Representative time courses of evoked responses in the oddball paradigms. The traces represent VEPs (left) and AEPs (right) from indicated recording sites **(B)** in the visual (#9) and **(C)** auditory cortices (#17). Prominent components of these traces, i.e., visual P1 (vP1), vMMN, auditory P1 (aP1), and auditory MMN (aMMN) are pointed. The time course of stimulus presentation is indicated at the bottom of the inset. **(D)** Statistical confirmation of MMN as a negative deflection in the deviant responses. The lines show significance level under the null hypothesis that deviant responses are larger than standard or control responses at a given post-stimulus latency time (Wilcoxon one-sided rank-sum test with Bonferroni correction for 450 comparisons). The blue and red lines indicate significance levels for comparison of deviant and standard responses, and the light blue and pink lines indicate those for comparison of deviant and control responses. The blue and light blue lines indicate the recording sites in the visual cortex, and the red and pink lines indicate the recording sites in the auditory cortex. Horizontal broken lines indicate *p* = 0.05. **(E)** The amplitude of (i) vP1 in the standard, deviant, and control responses, and (ii) the negative deflection or MMN quantified in the subtraction of deviant responses from the standard or control responses. Dots indicate the mean amplitudes in the visual cortex of each animal and for each stimulus. Asterisks indicate statistical significance in *post hoc* analysis: **p < 0.01; ***p < 0.001 (Wilcoxon one-sided signed-rank test with Bonferroni correction for three comparisons following the Kruskal-Wallis test).

We then mapped single-modal MMNs, i.e., vMMN and aMMN, and tested whether vMMN exhibited deviance-detection properties. Figure 3A shows the mapping of VEPs and AEPs recorded in the visual oddball, visual many standards control, and auditory oddball paradigms. Again, the first positive peaks, i.e., vP1 and aP1, appeared only in the visual and auditory cortices, respectively, and aP1 appeared earlier than vP1, as described above (Figures 3B,C and Supplementary Figure 1). In contrast, the deviant responses in both regions exhibited a significant negative deflection with a longer latency than each P1; in other words, vMMN appeared in the auditory cortex, and aMMN appeared in the visual cortex, without distinct P1s. In addition, as shown in Figures 3B-D, vMMN appeared earlier in the visual cortex than the auditory cortex, and aMMN appeared earlier in the auditory cortex than the visual cortex, suggesting propagation of single-modal MMN toward another sensory cortex.

Thereafter, the visual “many standards control” paradigm was applied to test the deviance detection property of vMMN. The control responses did not exhibit negative deflection as seen in the deviant responses (Figures 3A-C). The results also confirmed that the negative deflections in the deviant responses were significantly larger than those in the standard and control responses (Figure 3D). Additionally, comparison of the amplitude of vP1, i.e., the maximum potential within 200 ms from the stimulus onset, demonstrated clear adaptation in the standard responses (Figure 3Ei, Kruskal-Wallis test, *p* = 0.0052; *post hoc* Wilcoxon one-sided signed-rank test with Bonferroni correction for three comparisons, *p* = 0.0016 for standard vs. deviant responses, 0.00036 for standard vs. control responses). Conversely, amplitude of the negative deflection, i.e., maximum of the difference wave between 50 and 450 ms from the stimulus onset, did not differ irrespective of whether the deviant response was subtracted from the standard response or from the control response (Figure 3Eii, *p* = 0.63, Wilcoxon one-sided signed-rank test). Thus, vMMN in rats exhibits deviance-detection properties, as reported for aMMN in our previous study (Shiramatsu et al., 2013).

### Comparison Between Cross-Modal MMN and Single-Modal MMN

**FIGURE 4:**
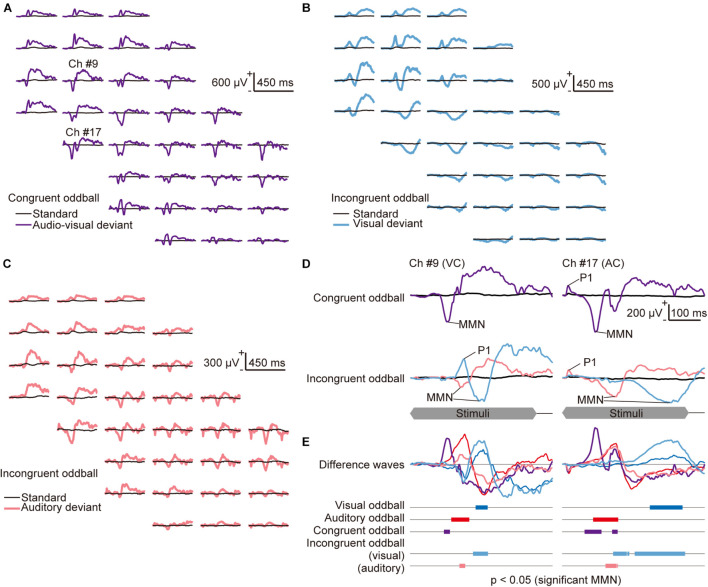
Mapping of the congruent and incongruent audio-visual mismatch negativity (avMMN). **(A–C)** Representative mapping of responses recorded **(A)** in the congruent audio-visual oddball paradigm, where the auditory and visual deviant stimuli were always presented together (purple lines), and (**B–C)** in the incongruent audio-visual oddball paradigm, where **(B)** the visual deviant (light blue lines) and **(C)** the auditory deviant (pink lines) were delivered independently. Both incongruent oddball paradigm maps show the same standard (black lines) responses. **(D)** Responses in the congruent oddball (top) and the incongruent oddball (bottom) paradigm from the indicated recording sites in the visual (left, #9) and auditory cortices (right, #17). The time course of stimulus presentation is indicated at the bottom of the inset. **(E)** Difference waves obtained by subtracting the deviant responses from the standard responses in all tested oddball paradigms: visual oddball (blue lines), auditory oddball (red lines), and congruent audio-visual oddball (purple lines). In the incongruent audio-visual oddball paradigm, the difference wave between the standard, visual-deviant (light blue), and auditory-deviant responses (pink) are shown separately. The bars in the bottom inset represent time courses of each MMN, i.e., the latencies when significant differences were found under the null hypothesis that deviant responses are larger than standard responses (Wilcoxon one-sided rank-sum test with Bonferroni correction for 450 comparisons).

Mapping of the cross-modal MMN revealed putative cross-modal effects on deviance detection. In response to audio-visual deviant stimuli in the congruent oddball paradigm, early P1 appeared in the auditory cortex, followed by a negative wave in both the visual and auditory cortices (purple lines in Figures 4A,D and Supplementary Figure 1). Relatively slow P1 in the visual cortex, which is similar to vP1, was often absent; therefore, we often obtained responses similar to those seen in the auditory oddball paradigm. In the incongruent oddball paradigm, the visual deviant and auditory deviant responses were similar to the deviant responses obtained in the single-modal oddball paradigm (light blue and pink lines in Figures 4B-D). There were distinct P1 and earlier MMN in the same modality sensory area as the deviance and late MMN in the other sensory areas. The representative difference waves in all tested oddball paradigms showed that the vMMN in the single-modal visual oddball paradigm (blue lines in Figure 4E) and MMN in the incongruent oddball paradigm (light blue lines) were similar and that the aMMN in the single-modal auditory oddball paradigm (red lines) and MMN in the incongruent oddball paradigm (pink lines) were similar (Figure 4E). The latency in which a significant MMN (*p* < 0.05 in the comparison between the deviant and standard responses) was found was earlier in the auditory oddball, in the congruent oddball, and auditory deviance in the incongruent oddball than in the visual oddball and visual deviance in the incongruent oddball.

**FIGURE 5:**
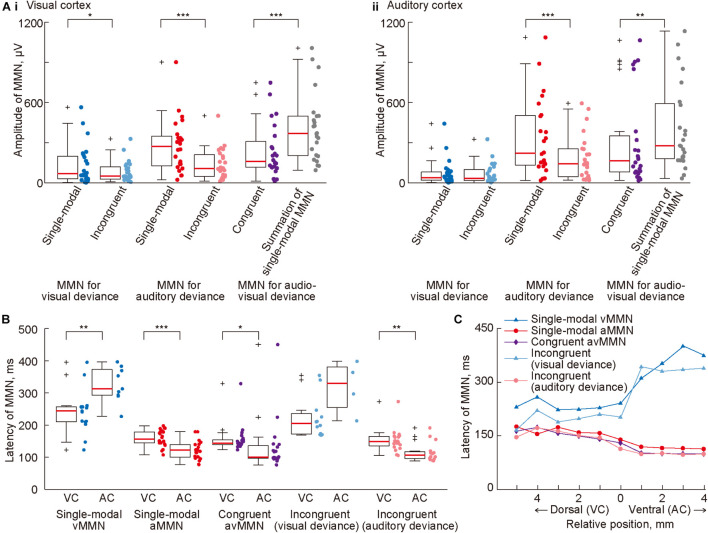
Cross-modal effect on mismatch negativity (MMN) amplitude and latency. **(A)** Mean amplitude of MMN in (i) the visual and (ii) auditory cortices, quantified in all tested paradigms. For comparison, the sum of vMMN and aMMN in the single-modal oddball paradigm is also shown (rightmost). **(B)** Comparison of mean MMN latency in the putative visual cortex (VC) and the putative auditory cortex (AC). **(C)** Propagation of MMN. The median latency of MMN in each row of the recording site was plotted with respect to the relative vertical position from the most ventral row of the putative visual cortex. In panels **(A,B)**, dots indicate the mean amplitudes of MMN in each putative region in individual animals. Asterisks indicate statistical significance: *p < 0.05; **p < 0.01; ***p < 0.001 (Wilcoxon one-sided signed-rank test).

We further investigated the cross-modal effect on MMN amplitude using pooled data. A simple test of the cross-modal effect is to compare MMN amplitude in the cross-modal paradigm with MMN assumed to be elicited independently in each sensory modality. If there is no cross-modal effect and vMMN and aMMN are always elicited separately, then the amplitude of the MMN for the visual or auditory deviance in the incongruent oddball paradigm (light blue and pink dots in Figure 5A) should be the same as the amplitude of the vMMN or aMMN in the single-modal oddball paradigm (blue and red dots), respectively. Moreover, the amplitude of the avMMN in the congruent oddball paradigm (purple dots) may be the same as the summation of the vMMN and aMMN in a single-modal oddball paradigm (gray dots). We found evidence that did not support the above hypothesis. The amplitudes of MMN in the incongruent oddball were smaller than those in the single-modal oddball paradigm in some cases, and the amplitudes of avMMN in the congruent oddball were smaller than the summation of the MMN amplitude in the single-modal oddball paradigm (Figure 5Ai: *p* = 0.018, vMMN in single-modal oddball vs. MMN for visual deviance in incongruent oddball; *p* = 7.7 × 10^–5^, aMMN in single-modal oddball vs. MMN for auditory deviance in incongruent oddball; *p* = 0.00014, avMMN in congruent oddball vs. summation. Figure 5Aii: *p* = 0.00012, aMMN in single-modal oddball vs. MMN for auditory deviance in incongruent oddball; *p* = 0.0017, avMMN in congruent oddball vs. summation; Wilcoxon one-sided signed-rank test), indicating a cross-modal effect on deviance detection.

In analysis of the peak latency of MMN, it was obtained as the post-stimulus time when the amplitude of the MMN was quantified as the maximum and significant potential difference between 50 and 450 ms from the stimulus onset. The latency pattern of avMMN in the congruent oddball paradigm (purple dots in Figure 5B) resembled closely with that of aMMN (red dots) as compared to the latency pattern of vMMN (blue dots), indicating the advantage of aMMN over vMMN. Comparison of MMN latency between the visual and auditory cortices showed two types of generation and propagation of MMN. First, vMMN in the single-modal oddball and the MMN for visual deviance in the incongruent oddball (blue and light blue dots) were generated in the visual cortex and propagated with longer latency. Second, aMMN in the single-modal oddball, the MMN for auditory deviance in the incongruent oddball, and avMMN in the congruent oddball (red dots, pink dots, and purple triangles) were generated earlier in the auditory cortex and propagated to the visual cortex (Figure 5B: *p* = 0.0020 and 0.00017, vMMN and aMMN in the single-modal oddball paradigm; *p* = 0.019, avMMN in the congruent oddball paradigm; *p* = 0.0017, MMN for the auditory deviance in the incongruent oddball paradigm; Wilcoxon one-sided signed-rank test). Pooling the data according to the relative vertical distance from the border between the visual and auditory regions made these two types of propagation very clear (Figure 5C). The propagation time, i.e., the latency difference between the areas was 80–130 ms from the visual to the auditory area and 35–45 ms in the opposite direction. Taken together, these results strongly suggested that visual and auditory deviance detection did not work independently under the cross-modal oddball paradigm. Additionally, cross-modal MMN responding to single-modal deviances was mainly mediated by the corresponding sensory area, whereas avMMN responding to congruent deviance appeared to have a robust source in the auditory area.

### Pharmacological Effect of NMDA Receptor Antagonist Administration in the Visual Cortex on Each MMN

**FIGURE 6:**
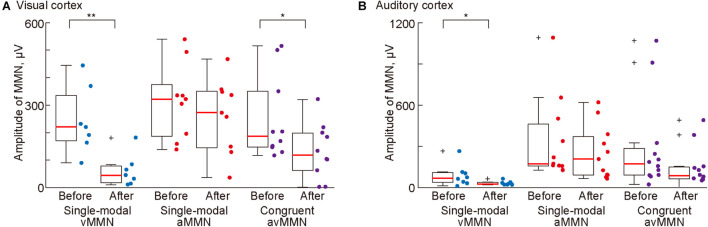
Pharmacological effects of administration of an *N*-methyl-D-aspartic acid (NMDA) antagonist (AP5) in the visual cortex on mismatch negativity (MMN). Mean amplitude of MMN in **(A)** the visual and **(B)** auditory cortices before and after administration of AP5 to the visual cortex. Dots indicate the mean MMN amplitudes in each putative region in individual animals. Asterisks indicate statistical significance: *p < 0.05; **p < 0.01 (Wilcoxon one-sided signed-rank test).

Finally, we tested whether NMDA receptor antagonist administration attenuated single-modal vMMN and aMMN and cross-modal avMMN in the congruent oddball paradigm. For this analysis, data were included only when the deviant responses that were obtained before placing the agarose gel sheet exhibited a significant MMN. The antagonist caused significant reductions in the mean amplitude of single-modal vMMN in both sensory areas (Figure 6; *p* = 0.0012 and 0.025 for the visual and auditory cortices, respectively; Wilcoxon one-sided signed-rank test) but not of single-modal aMMN (*p* = 0.33 and 0.17 for the visual and auditory cortices, respectively). These different changes indicated that the agarose gel sheet allowed administration of AP5 to the visual cortex.

Lastly, we investigated whether avMMN elicited in the congruent oddball paradigm is mediated by the visual cortex. The mean amplitude of avMMN in the visual cortex was significantly reduced (*p* = 0.038, Wilcoxon one-sided signed-rank test). This reduction in mean amplitude was not significant in the auditory cortex (*p* = 0.056); however, the maximum amplitude in this area was significantly reduced after application of the NMDA antagonist (*p* = 0.027, data not shown). Taken together, these results show that NMDA receptor blockade in the visual cortex attenuated vMMN in the single-modal oddball paradigm and avMMN in the congruent oddball paradigm.

## Discussion

In this study, we investigated whether and how cross-modal information processing affects MMN in rodents. After using a surface microelectrode array to map vMMN and aMMN, we found that vMMN in rats exhibited characteristics similar to those previously reported for aMMN—a negative deflection following the P1 response, the deviance detection property, generation from the corresponding sensory area, and dependence on NMDA receptors in that area (Shiramatsu et al., 2013; Shiramatsu and Takahashi, 2021). Furthermore, we recorded three types of cross-modal MMN, that is, avMMN in the congruent oddball paradigm and MMN for the visual and auditory deviances in the incongruent oddball paradigm. Mapping of the amplitudes and latencies of the tested MMNs and administration of an NMDA blocker showed cross-modal effects on MMN. To date, cross-modal audio-visual MMN in rodents has not been reported. Our results emphasize the importance of rodents as animal models for MMN study, and future studies on the neural mechanisms combining multisensory integration and deviance detection are expected to provide electrophysiological evidence to confirm the links between MMN and predictive coding theory.

### Functional Similarity of vMMN Between Rodents and Humans

This study demonstrated four functional characteristics of rat vMMN that were comparable to those of human MMN: morphological characteristics, the deviance detection property, generation in the corresponding sensory area, and dependence on NMDA receptors. First, vMMN appeared in the deviant responses as a negative deflection following vP1 responses, as reported in humans (Sams et al., 1985; Tiitinen et al., 1994; Amenedo and Escera, 2000; Näätänen et al., 2007). The peak latencies of vP1 and vMMN in the visual cortex were approximately 90 and 110–400 ms, respectively (Figures 3A,B, 5B). These latencies were longer than those of the auditory responses, i.e., aP1 at 20 ms and aMMN at 70–190 ms, as reported in several physiological studies (Meredith et al., 1987; Bell et al., 2006; Jaekl et al., 2014), yet the latencies of human vMMN and aMMN are comparable, i.e., 120–300 ms (Berti and Schröger, 2001; Czigler et al., 2002; Pazo-Alvarez et al., 2003; Näätänen et al., 2007). One possible reason is the weak eyesight of rats as nocturnal animals, which sometimes exhibits different structures of the visual cortex compared to humans (Kondo et al., 2016; Maruoka et al., 2017). Our supplemental results indicated that vMMN was not sensitive to the magnitude of deviance (Supplementary Figure 2; there was no increase in the amplitude of vMMN for more distant deviants), which also supports the possibility of rats having different visual deviance detection from that of humans. Another possibility is that the visual stimuli used in this study induced weak activation in the rat visual cortex or its deviance detection system (Alho et al., 1992; Czigler et al., 2002; Pazo-Alvarez et al., 2003). A previous mice study reported vMMN in a similar time course with humans (Hamm and Yuste, 2016), using full-field square-wave gratings, which might cause different activation from one vertical bar used in the present study.

Second, despite the long latency, vMMN in rats exhibited a deviance detection property as well as human vMMN (Czigler et al., 2002, 2006; Pazo-Alvarez et al., 2004; Kimura et al., 2009). It has been claimed that both vMMN and aMMN in humans represent deviance detection and are not mere effects of adaptation, because they are also elicited by complex changes, such as a violation of categorization or sequential rules (Czigler et al., 2006; Astikainen and Hietanen, 2009; Chang et al., 2010; Clifford et al., 2010; Stefanics and Czigler, 2012; Czigler, 2014). The present study applied a previously designed control paradigm and distinguished the deviance detection property in the vMMN from adaptation. To date, this is the first evidence of the deviance detection property in vMMN in rats, following the previous reports in rabbits (Astikainen et al., 2000) and in mice (Hamm and Yuste, 2016).

Third, the present mapping technique across the two sensory cortices revealed that the MMN for the deviance in one sensory modality first appeared in the corresponding sensory cortex, consistent with several electroencephalography studies (Scherg et al., 1989; Csépe et al., 1992; Tiitinen et al., 1993; Alho et al., 1996; Berti and Schröger, 2001; Czigler et al., 2002). However, because of the low spatial resolution of the present microelectrode array, we could not identify the precise MMN-generating subregion in the visual cortex. It is expected that the higher-order visual area, i.e., the secondary visual area (V2L), or both the primary and higher-order visual areas, are involved, considering that aMMN is generated from the secondary auditory cortex in cats (Pincze et al., 2001) and spreads toward the belt area in rats (Shiramatsu et al., 2013).

Lastly, the present study also demonstrated that single-modal MMN is mainly mediated by NMDA receptors in the corresponding sensory cortex, indicating the role of NMDA receptors in the MMN generation process. Accumulating evidence from both clinical and animal studies has shown that NMDA receptors mediate aMMN (Kreitschmann-Andermahr et al., 2001; Umbricht et al., 2002; Tikhonravov et al., 2010; Shiramatsu et al., 2013), and that aberrant NMDA receptor function diminish vMMN (Shelley et al., 1991; Baldeweg et al., 2004; Urban et al., 2008; Farkas et al., 2015). To date, however, no previous study has directly shown the decrease in vMMN caused by the NMDA receptor blockade, or the effect of limited, local infusion of an NMDA receptor antagonist. The present invasive recording in the rodents allowed us to demonstrate that the blocker that was locally infused into the visual area reduced vMMN in the visual and auditory areas, while aMMN remained unaffected in both areas (Figure 6). These results indicate that NMDA receptors contribute to the neural process of deviance detection in a modality-specific manner. However, these receptors might not directly mediate the negative deflection itself. Taken together with a recent report that another neuromodulator, i.e., somatostatin, worked with a similar time-course of MMN (Hamm and Yuste, 2016), the deviance detection process should be divided into several sub-steps, with NMDA receptors contributing to the early steps, such as the construction of prediction. Future studies using local application of blockers or genetically engineered animals could identify the step-by-step role of each neuromodulator in the MMN generation process.

### Cross-Modal Effects on MMN in Rodents

The present study demonstrated cross-modal effects in the MMN elicited by three types of deviant stimuli, that is, congruent (or paired) audio-visual deviant and incongruent (or independent) visual and auditory deviant stimuli (Figure 1E). Cross-modal effects on the avMMN in the congruent oddball were demonstrated by three characteristics: the latencies similar to aMMN, dependence on the NMDA receptors in the visual area, and the non-linear relationship of its amplitude. First, the shorter latency of the avMMN in the auditory area (Figures 5B,C) indicates that some parts of the neural substrates of single-modal aMMN may also mediate avMMN. Second, after the local blockade of the NMDA receptor in the visual area, amplitudes of the avMMN and single-modal vMMN decreased, while that of the single-modal aMMN remained unchanged (Figure 6). This result pharmacologically demonstrated the contribution of the deviance detection system in the visual area to avMMN mediation. Lastly, the amplitude of the avMMN was not comparable to the summation of single-modal vMMN and aMMN, supporting a cross-modal effect on MMN (Figure 5A). When the deviant detection system manages double deviants independently, i.e., sound frequency and intensity, the amplitude of MMN shows a linear relationship, i.e., summation of the MMNs for corresponding single deviants (Paavilainen et al., 2001; Wolff and Schröger, 2001). Taken together with the previous report that multi-modal interactions between the deviance detection systems also exhibited non-linear MMN for double-deviants (Butler et al., 2012), the present results demonstrated cross-modal interaction between visual and auditory systems on avMMN in the congruent oddball paradigm.

In the incongruent oddball paradigm, smaller MMNs than the corresponding single-modal oddball paradigm also indicated cross-modal effect (Figure 5A). For this non-linear relationship, there were two possible mechanisms. When both modalities were considered together, the probability of deviants was twice (20%) of that of the single-modal oddball paradigm (10%), which could elicit a smaller MMN (Sato et al., 2000; Sabri and Campbell, 2001; Näätänen et al., 2007). The second possibility is that the impact of the deviance was different in the incongruent oddball paradigm due to the multimodal feature integration—when the paired stimuli were perceived as one audio-visual object, the change in the single-modal characteristic should be a “weak deviant,” possibly eliciting small MMN. Considering that even unconscious animal subjects can produce MMN based on empirically acquired information (Shiramatsu and Takahashi, 2018), the different impacts of deviance under multimodal feature integration should also affect MMN. In both cases, it can be assumed that the deviance detection functions in the visual and auditory systems were not independent but rather interacted with each other.

To date, audio-visual MMNs have been obtained in humans using experimental designs that highlight top-down effects, such as the McGurk-MacDonald illusion or pairs of specific languages, or language-replicated sounds with letters or speaking faces (Colin et al., 2002a, b; Saint-Amour et al., 2007; Froyen et al., 2008; Andres et al., 2011; Stekelenburg and Vroomen, 2012). Both stimuli need some knowledge of the corresponding language or the habituation process to integrate the appropriate auditory and visual pairs, emphasizing empirically acquired top-down effects on these MMNs. A few studies have used non-linguistic stimuli and demonstrated cross-modal effects on congruent avMMN and incongruent vMMN and aMMN (Stekelenburg et al., 2004; Horvath et al., 2013). However, one of the main interests was the ventriloquist illusion (Stekelenburg et al., 2004) and to demonstrate that MMN reflects our illusory perception; therefore, the detailed interpretation of the results differs from that in the present study. We believe that, to distinguish between top-down and bottom-up effects on audio-visual MMN for linguistic stimuli, further investigation of MMN in humans and animal models using simple stimuli, such as those employed in this study, will be beneficial.

### Possible Neural Mechanisms of the Cross-Modal Effect on MMN

The present cross-modal MMN can be modified in various processing stages, i.e., the bottom-up, corticocortical, and top-down pathways (Cappe et al., 2011). Several subcortical nuclei and thalamocortical projection in the auditory ascending pathway exhibit sensitivity to visual inputs and vice versa (Budinger et al., 2006; Alvarado et al., 2007; Porter et al., 2007; Henschke et al., 2015; Kimura, 2020). These subcortical nuclei are sensitive to oddball paradigm and often exhibit strong stimulus-specific adaptation (Escera and Malmierca, 2014; Shiramatsu et al., 2016a; Parras et al., 2017; Takahashi et al., 2020); therefore, they can convey cross-modal information about repetitive inputs to cortical areas. Direct crosstalk between sensory cortices can also affect ongoing predictions and deviance detection (Falchier et al., 2002, 2010; Rockland and Ojima, 2003; Clavagnier et al., 2004; Budinger et al., 2006). Lastly, top-down information about cross-modal integration is expected to influence cortical sensory processing. The functional areas for sensory integration are widely distributed in the brain, i.e., the prefrontal and parietal cortices (Romanski, 2007; Sereno and Huang, 2014). Top-down projections from these associative areas often terminate in higher sensory regions, which are putative foci of MMN generation (Alho et al., 1996; Romanski et al., 1999; Pincze et al., 2001; Shiramatsu et al., 2013). A previous study reported that damage to the prefrontal cortex affected MMN in the auditory cortex, which supports the hypothesis that such top-down projections contribute to the generation of cross-modal MMN to some extent (Alain et al., 1998). Thus, we can expect further advancements in microscale electrophysiological and pharmacological techniques in animal models to reveal the precise neural mechanisms underlying both cross-modal MMN and pre-attentive sensory integration.

As a new phenomenon that could be the subject of future investigation in cross-modal animal MMN, we found interregional propagation of MMN between the visual and auditory cortices (Figure 5C). However, the present study could not clarify whether this phenomenon was similar to stimulus-induced traveling waves or transmitted signals, such as the late frontal sources of human aMMN (Rinne et al., 2000). Traveling waves are characterized in multichannel recordings and mainly mediated by long-range horizontal fibers of intracortical axons, spreading within the superficial layers of the cortex (Muller et al., 2018). Involvement of the superficial layers can explain why robust propagation was only found in MMN but not P1—MMN is thought to reflect neural components in the superficial layers, while P1 mainly reflects synaptic current to cortical layer 4 (Javitt et al., 1996; Lee et al., 2004; Fishman and Steinschneider, 2012). Moreover, smooth surface of the rat cortex might emphasize such horizontal spread. The speed of this propagation was reported to be 0.1–0.8 m/s, which is not vastly different from the present results, i.e., 0.02–0.06 m/s (Figure 5C; vMMN and aMMN required 80–130 and 35–45 ms, respectively, for an approximately 2-mm propagation). Another possibility is that MMN in one sensory area is transmitted to another area, where it elicits a new MMN-like deflection. In this scenario, the variation in MMN latency between sensory areas should be more significant than within a sensory area, as seen in our study (Figure 5C). The asymmetrical transfer rate between visual-to-auditory and auditory-to-visual propagation also supports this possibility. As the poor spatial resolution of the current recording system prevented detailed mapping of latency, we cannot reach any definitive conclusions. However, in both scenarios, the propagation of MMN may provide cross-modal modulation in other sensory cortices by altering neuronal excitability.

### Methodological Considerations

Although the present study succeeded in simultaneous recording from the visual and auditory cortices of rats, there were certain limitations in the context of the comprehensive recording. First, it was often difficult to expose the entire visual cortex surgically. In such cases, the ventral and anterior parts of the visual-related area, which is assumed to include higher-order subregions (i.e., the V2L) than the primary subregion of the visual cortex, was used for recording. Second, due to the design of the microelectrode array, we often failed to cover the higher-order auditory cortex, that is, the ventral auditory subfield (Takahashi et al., 2004, 2005; Shiramatsu et al., 2016b, c). Since our focus was on the global trends between the two sensory cortices, we preferentially covered the boundaries of these areas rather than the more distant subregions. Therefore, we could not categorize the primary and higher-order subregions of these cortices and failed to reveal different cortical maps between P1 and MMN, as in our previous report (Pincze et al., 2001; Shiramatsu et al., 2013). Third, the large inter-electrode distance of 1 mm prevented us from identifying the precise audio-visual border, which made propagation velocities ambiguous. In the future, using a microelectrode array with a higher density of recording sites and a larger coverage area toward the outer boundary of the targeted sensory areas will provide more detailed electrophysiological evidence to elucidate the cross-modal interaction under audio-visual oddball sequences.

In the analysis for the incongruent oddball paradigm, the standard and deviant responses were not derived from the identical audio-visual stimuli (see section “Materials and Methods”). Although the compared standard and deviant stimulus should be identical, its influence on the present result is thought to be small from two perspectives. First, the standard responses did not show distinct deflection in the latency of MMN (Figures 3, 4); therefore, subtraction of the standard responses (almost zero potential) from the deviant responses would not affect the quantified amplitude of MMNs. Second, for the quantification of MMN, we chose the standard and deviant responses so that the stimuli of the deviant modality would be the same. The deviant stimuli would strongly stimulate the sensory system and trigger MMN, supported by the distinct P1 in the modality of deviance (Figure 4D); therefore, this subtraction was reasonable in the absence of an identical standard response.

### Future Directions

The present study provided evidence of cross-modal effects on animal MMN, which had not been described previously and which will inform future research in this area. Accumulating evidence indicates that MMN in animal models, particularly in rodents, could be homologous to human MMN. We also believe that future studies on rodent MMN will contribute to the elucidation of neural mechanisms underlying aberrant information processing in specific psychological disorders. We previously demonstrated that aMMN in rats reflects salience processing, based on individual experience, inspired by a “naive” asymmetry of the amplitude of aMMN between upward and downward changes (Shiramatsu and Takahashi, 2018). In the present study, vMMN also exhibited similar asymmetry between forward and backward shifts of the stimulus (Supplementary Figure 2 shows that forwarding changes elicited larger vMMNs than backward changes). This result suggests that vMMN in rats, as in human vMMN, also represents empirical salience (Sulykos et al., 2015). Taken together with the links between the small aMMN and aberrant salience processing in patients with schizophrenia (Baldeweg et al., 2004; Nelson et al., 2014), the present results raise the possibility that such aberrant salience processing can also develop in the visual domain, which could stimulate and inform further investigations into the general neural substrates of specific psychological disorders.

## Data Availability Statement

The raw data supporting the conclusions of this article will be made available by the authors, without undue reservation.

## Ethics Statement

The animal study was reviewed and approved by the Committee on the Ethics of Animal Experiments at the Graduate School of Information Science and Technology, the University of Tokyo.

## Author Contributions

TS, KM, KI, and HT designed the study and approved the final version of the manuscript. KM and KI performed the experiments. TS and KM analyzed the data, interpreted the results, and prepared the figures. TS drafted the manuscript. KM, KI, and HT revised the manuscript. All authors contributed to the article and approved the submitted version.

## Conflict of Interest

The authors declare that the research was conducted in the absence of any commercial or financial relationships that could be construed as a potential conflict of interest.

## Publisher’s Note

All claims expressed in this article are solely those of the authors and do not necessarily represent those of their affiliated organizations, or those of the publisher, the editors and the reviewers. Any product that may be evaluated in this article, or claim that may be made by its manufacturer, is not guaranteed or endorsed by the publisher.

## Footnotes

1
http://psychtoolbox.org/
