# Supplementary material for: Auditory, Visual, and Cross-Modal Mismatch Negativities in the Rat Auditory and Visual Cortices
Source: Front Hum Neurosci. 2021 Sep 17;15:721476. doi: 10.3389/fnhum.2021.721476 (PMC8484534; doi:10.3389/fnhum.2021.721476)
Supplement: Supplementary file 1 [file Data_Sheet_1.pdf]

Supplementary Materials

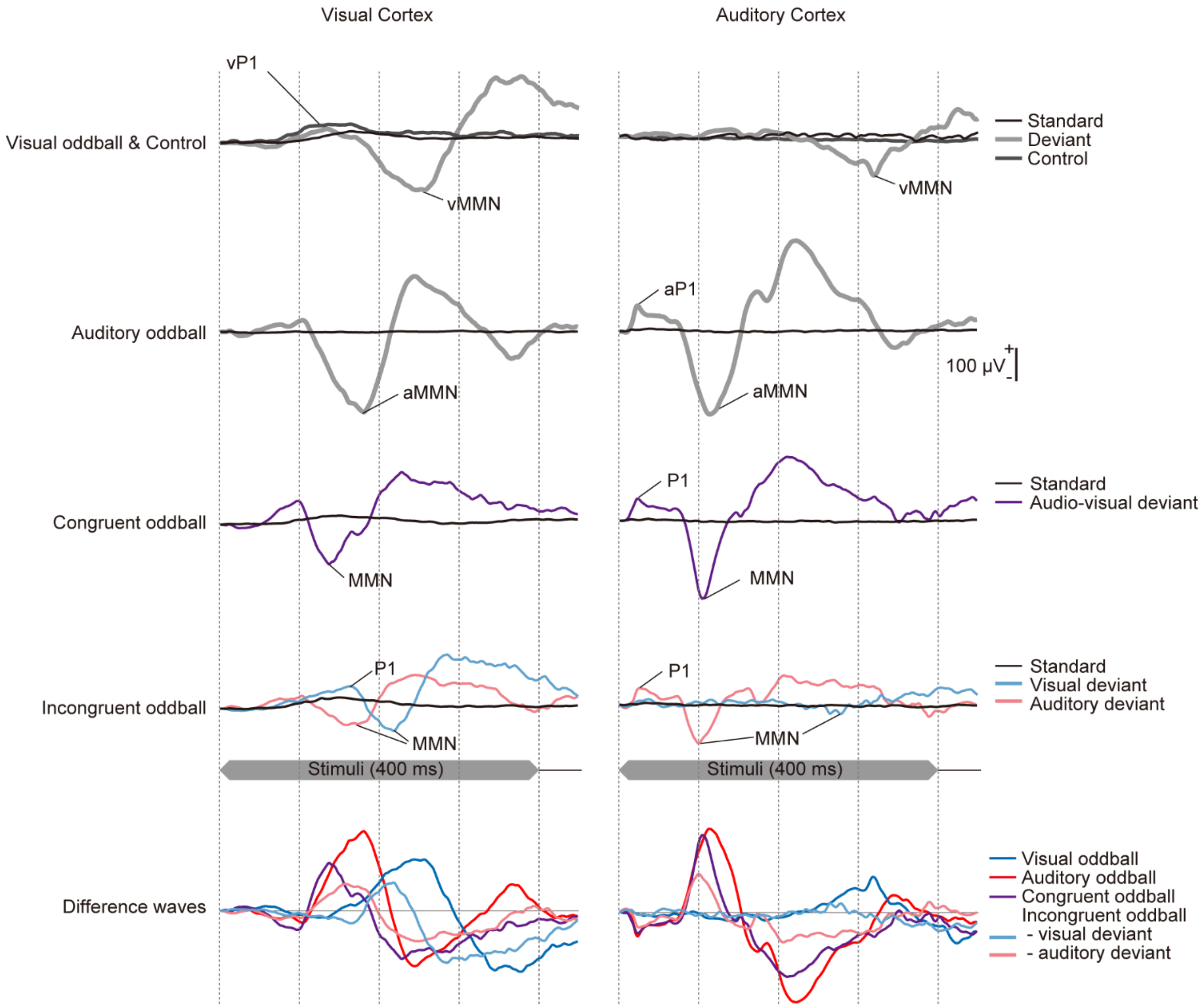

Supplementary Figure 1. Grand-average waveforms for all tested animals.

Grand-averaged waveforms for representative recording site in all tested animals. The traces represent standard, control (only in the visual oddball) and deviant responses from representative recording sites in the visual (left) and auditory cortices (right). Prominent components of these traces are pointed as Figure 3 and 4. The time course of stimulus presentation is indicated.

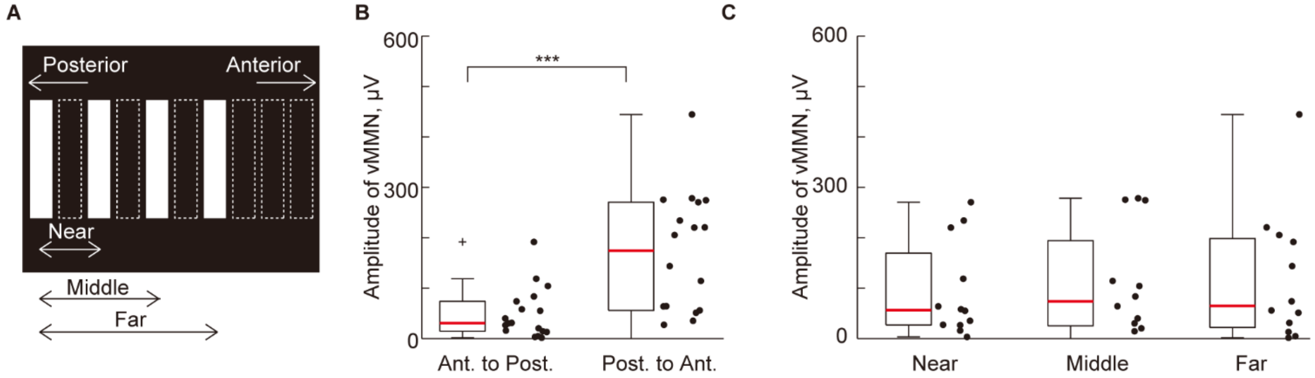

Supplementary Figure 2. Asymmetric property of visual MMN (vMMN)

(A) In six rats, we obtained vMMN using three pairs of visual stimuli. For each pair, two of the ten horizontally evenly spaced stimulus locations were used, i.e., first and third from the left for the "Near" pair, first and fifth for the "Middle" pair, and first and seventh for the "Far" pair. The "Far" pair was mainly investigated in the main study. Additionally, each pair elicited two types of MMN according to the change in direction from the standard to deviant stimuli. Because we presented these stimuli to the left eye of rats, a change from the left to right stimuli is assumed to be a "posterior to anterior" change or forwarding change and vice versa. (B) Comparison of mean amplitude in the visual cortex. The amplitude of vMMN was larger for the posterior to anterior changes ($p = 1.5 \times 10^{-4}$, Wilcoxon one-sided signed-rank test). (C) Kruskal–Wallis test revealed no significant difference of the amplitude of MMN between three groups of magnitude of deviance (Near, Middle, or Far; $p = 0.93$). Dots indicate the mean amplitudes of vMMN in the visual cortex in individual animals. Asterisks indicate statistical significance: ***, $p < 0.001$ (Wilcoxon one-sided signed-rank test).
